# Sprains

**Published:** 1877-03

**Authors:** 


### SPRAINS

Or strains of the joints are very painful, and more tedious of recovery than a broken bone. What we call flesh is *muscle;* every muscle tapers down to a kind of string, which we call cord or sinew. The muscle is above the joint, and the sinewy part is below it, or *vice versa*, and the action is much like that of a string over a pulley. When the ankle, for example, is "*sprained*," the cord, tendon, or ligament (all mean the same thing) is torn in part or whole, either in its body, or from its attachment to the bone, and inflammation—that is, a rush of blood to the spot—takes place as instantly as in case of a cut on the finger. Why? For two reasons. Some blood-vessels are ruptured, and very naturally pour out their contents; and second, by an infallible physiological law, an additional supply of blood is sent to the part, to repair the damages, to glue, to make grow together, the torn parts. From this double supply of blood, the parts are overflown, as it were, and push out, causing what we call "*swelling*"—an accumulation of dead blood, so to speak. But dead blood cannot repair an injury. Two things, then, are to be done, to get rid of it, and to allow the parts to grow together. But if the finger be cut, it never will heal as long as the wound is pressed apart every half hour, nor will a torn tendon grow together, if it is stretched upon by the ceaseless movement of a joint, therefore, the first and indispensable step, in every case of sprain, is perfect quietude of the part; a single bend of the joint will retard what Nature has been hours in mending. It is in this way, that persons with sprained ankles are many months in getting well. In cases of sprain, then, children who cannot be kept still, should be kept in bed, and so with many grown persons.

The "*swelling*" can be got rid of in several ways; by a bandage, which, in all cases of sprain should be applied by a skillful physician—otherwise, mortification and loss of limb may result. A bandage thus applied keeps the joint still, keeps an excess of blood from coming to the part, and by its pressure, causes an absorption of extra blood or other extraneous matter.

Another mode of getting rid of the swelling is, to let cold water run on the part injured for hours.

